# Surgical Observations, with Cases and Operations

**Published:** 1837-10-01

**Authors:** 


### SURGICAL OBSERVATIONS ON TUMORS, WITH CASES AND OPERATIONS. By *John C. Warren*, M.D. Professor of Anatomy and Surgery in Harvard University, and Surgeon of the Massachusetts General Hospital. 8vo. pp. 602. Sixteen coloured Lithographic Plates. Boston, 1837.

Dr. Warren justly observes that our knowledge with respect to tumors is still very vague, and our treatment of them is consequently unsatisfactory. The various attempts which have been made to classify them have failed to a greater or a less degree, and in no respect have all classifications failed so signally as in their relations to diagnosis.

The basis of the diagnostic signs of tumors must necessarily rest on their physical qualities. These are either primary or secondary. The former are such as are common to all bodies—figure, solidity, extension, situation. The secondary qualities are colour, sound, the sensation they occasion, and so forth. Unfortunately it happens that the primary qualities are those only which are invariably present, and they are so common in kind to all tumors, that they cease to afford very useful marks of discrimination. A decidedly malignant tumor, for example, may differ little, or not appreciably, from a non-malignant one, in form, or size, or hardness. Nor are the secondary or accidental qualities so constant or so peculiar as to supply the want of definite primary ones. One malignant tumor for example may have a mottled bluish colour on the surface, from the development of its venous system—but another tumor of the same nature may be deficient in this characteristic.

Such circumstances as these have operated to produce that uncertainty and confusion which still prevail with regard to the discrimination of tumors. Our knowledge is, no doubt, much greater than it was, for the close attention which has been directed to the subject has dispelled many erroneous notions, and established many valuable general conclusions. What has been done has been the result of the patient observation of facts, and what is to be done must be effected in the same manner.

We agree with Dr. Warren in the following opinions :—

"The difficulties arise from a number of causes: from the great variety of these diseases, which is such that the most experienced surgeon is frequently meeting with species he has never before seen; from the resemblance in external appearance between tumours whose character is quite different; from the want of an arrangement which may enable him to view them in groups, instead of being compelled to consider them, as often happens, merely as individuals.

These difficulties in the actual state of our data are insuperable. The last has been frequently the subject of the labours of ingenious and experienced men. Attempts have been made at arrangement and classification; but with so little success, that even the authors of these attempts have risen from their labours with disappointment and despair.

The difficulties of diagnosis are not the greatest which attend this subject. We are in ignorance of the causes of these morbid changes; of their intimate texture and organization; and in many instances of their diversity from or identity with that of the textures in which they are situated. More facts are wanted. More histories of the origin, course, and results of such affections. More observations on their intimate structure at the different periods of their existence.

You will not therefore expect a complete and satisfactory account of these affections. What you may principally hope for is, a collection of a considerable number of objects, which being brought as it were into a single view, may, by means of a comparison of their appearances and symptoms, enable you to acquire some general notions in regard to them, and form some arrangement, which, however imperfect, is better than the confusion of an indiscriminate grouping." 3.

We are sorry to find that the system of medical polity in America does not operate to the facilitating of scientific enquiries. The division of labour which obtains in this country is the evidence at once of its wealth and its civilization, and those who rail at it are ignorant of the wants and the tendencies of society. But we will present Dr. Warren's admission of the working of the American system.

"Practical men in this country, *called as they are to every kind of medical duty,* possessing no time they can devote to study, but what is robbed from hours which the wearied faculties require for repose, can estimate the obstacles to any literary labour among us."—*Pref.* viii.

The work is divided into fourteen sections. They are in order, on epidermoid tumors; on dermoid tumors; on tumors of the cellular membrane; on muscular tumors; on periosteal tumors; on osseous tumors; on tumors of the glands; on tumors of the secreting glands; on tumors of the testis; on tumors of the mucous glands; on tumors of the vascular texture; on tumors of the membranous textures; on encysted tumors; on abdominal tumors.

It is obvious that by adopting such a method of arrangement as this, the same *kind* of tumor will be considered again and again, as it happens to occur in various parts. But although it is opposed to a scientific analysis of the varieties of morbid growth, it has the practical merit of presenting to the mind tumors as they occur in nature, and in offering us a sort of substitute for bed-side experience, or, rather, a clinical report of it. It precludes, too, the necessity for any analytical operations on our parts, and enables us to pick out what we think most interesting or most instructive.

The following remarks on the method of examining tumors are judicious. They shew an exact mind, and, what is quite as good, a candid one.

"Under the disadvantages from a want of information on a number of important points, we are limited in our means of distinguishing the various tumors from each other, with a satisfactory degree of confidence. To accomplish our diagnosis as far as practicable, we should begin the investigation with the acquirement of a history of the origin and progress of the tumour, its influence on the part where it is situated, and on the whole system. After having obtained this history, a physical examination should be conducted with great care, and if there be any obscurity in the case, should be repeated frequently, and under different circumstances. With the exception of steatomatous and simple glandular tumours, how few of these diseases appear on dissection as the imagination of the surgeon had depicted them. Such misconceptions do not prove either a want of attention or of skill. They prove only the uncertainties of the best-studied investigations, and the necessity of bringing to our aid all the methods of elucidation, which time, reflection, and care may furnish. Therefore we should never advise nor undertake any decisive measure, at first view, in any but the plainest cases. To be called to operate on a chronic tumour at first sight, is an impropriety which no prudent surgeon should submit to.

The physical qualities of tumours, like those of other bodies, are primary and secondary. Each of these, as far as may be, should be successively studied. The *figure* is the quality which first strikes our senses, and should be well considered. Certain species of tumour affect particular forms; the steatoma, for example, is commonly rounded, and encysted tumors always have this form. Bony tumors and those of the complicated glands are irregular. *Extension* is an important quality, as it involves the connexion with surrounding parts. This is more difficult to understand, because we have access to a part only of the object inspected, the rest being concealed by organs attached to it. The extent of the part beyond the reach of the senses of touch and vision, is that which may implicate the most important structures, and cause the greatest embarrassment. The *solidity* will decide whether the tumour consists of bone, as an exostosis; or of bone and soft substance combined, as an osteo-sarcoma; or of fluid, as in the encysted tumour. Of the secondary qualities, that of *colour* has a great influence on our judgment in these cases. When the natural colour of the skin over a tumour is changed to red, our first impression leads to the suspicion of a malignant action, which may be confirmed or confuted by further examination. If this change appeared in a chronic affection of the breast, the suspicion would probably be well founded; if on one in the back, the appearance would, most likely, prove to be accidental. We are perhaps ready to attribute too much importance to this quality, considering its variableness, and the different lights in which it appears to different persons. A lady came to me from the country with a very tumid breast, the skin of which was of a bright red colour, giving a formidable and very malignant aspect. On the following day the colour was less, and gradually diminished, till the part had the usual appearance of healthy skin, and the disease proved not to be malignant.

Besides these qualities, there are others which may be called accidental, such as *pulsation, vibration, undulation.* These may exist in the tumour itself, or in a part connected with it, and may indicate aneurism, aneurism by anastomosis, a fluid contained in a cyst; or the proximity of such diseases to the part inspected." 11.

In general, we believe that the best method of investigating cases is by first ascertaining the nature of the phenomena which actually exist, and subsequently enquiring into their history. The advantage of doing so is obvious. By knowing what there *is*, we have a clue to our inquiries into what there *has been*. If we attempt to obtain a history without any distinct object in view, we are apt to get a tedious and an inconclusive one, for patients commonly dwell on trivial and very often on fallacious circumstances. In the cases of tumors a better and a less rambling history is usually obtained, for being objects of sense, their rise and progress have generally been noticed with tolerable accuracy.

### I. Epidermoid Tumors.

We see nothing in this section to detain us.

### II. Dermoid Tumors.

Under the head of Dermoid Tumors, our author considers Lepoides—Keloides—and Eiloides.

1. *Lepoides.** This name is applied to the scaly crust, or bark-like roughness of the skin, seen on the face of old persons. The scale of the cutis, observes our author, is an irregular rough crust, of a brown colour, which might be mistaken for a wart. Crusts are very common in the face, in those advanced in life, and sometimes spread over a considerable surface, forming a rough brown-coloured plate; but the true lepoides is a circumscribed crust, which at first appears like a discoloured speck or a piece of earth. This falls off, and is renovated in the same form, for many successive years. When the crust separates spontaneously, it sometimes leaves a new cuticle behind, which gradually thickens. In other cases the cutis, being exposed, presents a spicular surface of unfavourable aspect. This surface throws out an ill-conditioned pus, which dries in the form of irregular scales. At length, owing to accidental irritation, or a change in the constitution, from age or other cause, the cutis ulcerates slowly, and forms an incurable sore or cancer of the face. The tendency of this disease is to advance very gradually without much pain, and without contaminating any parts, but those in contact with it; so that the patient may live a number of years before he is worn out by it. Thus the complaint is intermediate between the true malignant and the simple tumors. It is not absolutely malignant, for the constitution and distant organs are not contaminated. It is not simple, for it does not admit of cure. The only remedy is extirpation by the knife, or destruction by caustic. The indications for the operation are thus laid down by Dr. Warren.

"The mere existence of a scale on the face, even of a patient advanced in life, would not call for an operation. We should say, that this should be re-

---

sorted to when the following conditions are combined; a crust of small size, with a decided elevation and well-defined red edge; with pain or shooting; and any surrounding induration. These appearances would derive additional importance from old age. Should ulceration actually exist, the proper course will be more distinct; and this may be followed with a hope of success, even when ulceration is much extended, because the morbid action is limited to parts in immediate contact with the ulcerous affection." 28.

The best caustic, should the patient object to the knife, is the potassa cum calce. It should be applied through a hole of appropriate size cut in sticking-plaister. The paste must be employed to the thickness of the twelfth of an inch, and removed on the day after its application. The eschar separates in five or six days.

2. *Keloides.*—This name, objectionable on several accounts, was adopted by Alibert, from the red vascular processes, extending from the tumor, and giving it the appearance of crabs' claws.

Under the head of Keloides, Dr. Warren places three varieties. A. A white permanent elevation of the skin. B. The spider-like pimple of the face. C. The keloides of Alibert.

A. The first variety is a rising of the true skin, in the formof the cicatrix of a burn. It is a white elevation of the skin, without discolouration, or with a very slightly coloured margin; not sensible nor painful; but differing from the cicatrix of a burn in its disposition to grow, and in the difficulty of eradicating it. No ulceration occurs, nor does it terminate in a malignant disease. As it resembles the scar of a burn, so it sometimes originates in such a scar. It is seen to occur in disordered habits, after operations for the removal of small tumors, such as common warts, small encysted tumors, &c.

Such is the account given of this sort of tumor by our author. He relates two cases. One is sufficient for the illustration of the preceding description.

*Case.*—" A young lady shewed me a wart-like tumour on the right shoulder, which she had noticed two years before, in size about a quarter of an inch, and raised from the surface one-sixteenth of an inch. It was cut out; the wound healed slowly. In about four weeks it began to rise, and shortly attained a diameter of three-quarters of an inch, with less elevation than at first. Caustic potass was then applied. The eschar separated, and the wound was subjected to frequent touches of the nitrate of silver. After it healed, the raised cicatrix was again seen. I removed it once more by the knife, including a quarter of an inch of sound skin around, and a layer of cellular membrane from beneath. This time it healed well, and there was no return of the tumour." 42.

B. The second variety, continues our author, is a small red pimple, appearing most commonly on the face; radiating red processes into the surrounding skin. The size of the body of this tumor is usually about the eighth of an inch; the rays sometimes extend half an inch around it. For a long time after its existence, there is no pain nor any uneasy sensation. After having continued for years, it assumes a prickling and, finally, a burning pain; increases in size, ulcerates, and assumes a cancerous character. It is, perhaps, exclusively in the decline of life, and in free livers, that this tumor ulcerates. It should be extirpated early.

C. The third variety,—we follow Dr. Warren,—is a more troublesome and dangerous disease. Like the first, it resembles, in the early stage, the scar of a burn. In colour it is more red than the surrounding skin, and shoots out red vascular processes, which give it the crab-like form. The elevation is not great in the common form of the disease. It is sensible to the touch, and is attended with a burning pain. The parts which are most frequently its seat, are the back, shoulders, and neck. On dissection it exhibits a white fibrinous appearance, much like the scirrho-cancerous deposit in the female breast. The most remarkable fact about it is the disposition to regenerate after removal. This may happen a number of times; and eventually it may ulcerate, and become a malignant sore.

Dr. Warren relates a case of this "true keloides." Two operations were performed, and after each the tumor was regenerated. At each of the points, even where the suture needle was passed through the skin, there was a little rising, similar to the original, about the size of a bean, and quite red. He relates also a case where the keloid tumor was not supplied with any of the processes, which have been the means of its being likened to a crab.

3. *Eiloides.*—"The name we have given to this tumour is derived from the rolls and coils it forms. It is a very rare disease, and I have seen but one instance of it. This is a true production of the cutis, and continues so throughout its growth, although it may become very large. The first appearance of it is an elevation of the skin, similar to that from a burn; without pain, redness, heat, or ulceration; it increases to a great size, and ultimately affects the patient's health. If removed, it is reproduced. No cause is known, but a scrophulous habit seems to predispose to it. The specimen I present to you is of this description of disease. It appears like a triple coil of inflated intestine, each roll being four inches long, growing by a narrow base from the side of the neck. The patient, as you perceive by the colour of the skin, was a negress. She was about 15 years old when she first applied to me. I was astonished at the view of this remarkable production, hanging from the side of the neck. She had not been perfectly healthy, nor was she apparently much disordered. I proposed, and soon after executed the removal; taking care in the operation to include, as you see, a portion of the circumference of sound skin. Although the tumour was thus large, the edges of the skin could afterwards be approximated and united, in a great part, by the first intention. The non-united part suppurated and granulated; and during the healing process was frequently subjected to the action of astringent caustics. In four weeks the wound was healed perfectly smooth. About eighteen months after she came to me with a tumour in the same place, not so large as the first, but occupying a broader base. She wished to have it removed; and on my declining, applied to another practitioner, who operated on it. Soon after it regenerated; grew rapidly; the patient's health failed, and she died dropsical. On examination of the body, the lymphatic glands in the abdomen were greatly increased, forming large tumours throughout the cavity. The liver was diseased. The serous cavities and the cellular membrane were full of water. The case of Eleanor Fitzgerald, depicted in Mr. John Bell's Principles of Surgery, seems to have been of the same character as this." 49.

### III. Tumors of the Cellular Membrane.

On common steatoma, or fatty tumor, it is unnecessary, we imagine, to dwell. But when such tumors form beneath the muscles, or between them, the diagnosis becomes difficult, and their removal troublesome. Dr. Warren relates an interesting case of this description.

*Case.*—"Mr. N., a gentleman 30 years of age, very corpulent, presented himself at the M. G. Hospital, with a tumour at the angle of the ribs, on the right side, occupying a space apparently about five inches in diameter; not distinctly circumscribed; becoming flat on raising the arm, so as to excite a doubt whether it was any thing more than the accumulation of common adipose substance, or whether it might not be a fluid. It had been discovered about three weeks before I saw it. An incision being made through the skin, the adipose substance was dissected through, without any appearance of tumour. The latissimus dorsi muscle being exposed, was divided; yet no tumour discovered in nor under it. I began to apprehend that I had been mistaken; and some degree of excitement was experienced by those who witnessed the operation, at the difficulty. The serratus major muscle having been brought into view, was in its turn divided, and under this the tumour was found, buried in a mass of fat. A very firm adhesion to the ribs and intercostals, rendered it difficult to raise it from its attachment. When removed, it was found to be about an inch thick, and three inches diameter, composed of adipose substance, excepting in the middle, where was found a small cellular sac, containing a curdy fluid. The patient had a slight pleuritic attack three days after the operation. This being relieved by two bleedings, he did well afterwards." 57.

Mr. Lawrence has described, in the Medico-Chirurgical Transactions, a tumor of the cellular membrane wholly free from fat. Dr. Warren has witnessed no instance of it.

But he has seen in the cellular membrane a tumor composed of that tissue, condensed, and exhibiting a fibrous arrangement. It is much firmer than the adipose tumor, not so regular, being flattened, apparently by the action of muscles between or under which it is situated. It is not painful; causes much lameness, when in the extremities; and is apt to be regenerated, probably from prolongations, which escape detection at the time of operation. The colour is whitish, consistence firm, and texture laminated.

A case of this description occurred to our author in July, 1834. The tumor was about four inches in diameter; of a flattened form; was situated under the hamstring muscles, where the tendons separate. The dissection was deep and difficult, from the irregular processes running under the muscles; but the whole of it was removed apparently; the wound healed by the first intention, and the patient left town in a week from the operation. About a year after, that is, in the Summer of 1835, she returned with a tumor not quite so large, nearly in the same situation, which Dr. Warren again removed. The patient soon recovered, went home, and has not been heard of by our author since.

We observe nothing new in reference to the painful subcutaneous tubercle—a tumor which is usually more or less connected with a subcutaneous nerve.

### IV. MUSCULAR TUMORS.

These, observes Dr. Warren, are formed in the substance of the muscles. They are not very common. On external examination they are less distinctly defined than steatomatous tumors, and less moveable. When the muscles in which they are situated are perfectly relaxed, they possess a considerable mobility; when these are firmly contracted, the tumor is quite fixed,—and these circumstances constitute their most remarkable character. The diseased part is not very easily distinguished from the healthy; so that in operation it is necessary to trench deeply into the surrounding muscle. They are more frequently accompanied with pain than cellular tumors; and more disposed to degenerate into malignant affections. Their origin is often traceable to accidental injury,—a blow, strain, or continued pressure. After removal they are apt to re-appear. So far as we have seen, the muscular tissue would appear to be prone to fungus hæmatodes, or medullary tumors. We have seen many such cases, and in several the origin of the tumor was referred to some sudden exertion, which may be conceived to have ruptured or strained the muscular fibre.

The affections noticed by our author, are,—encysted muscular melanosis,—scirrhous muscular tumor,—malignant melanosis,—and fungus hæmatodes. These do not require much consideration at our hands, as we do not observe any very striking features in the cases. Those which deserve attention are the following.

1. *Encysted Muscular Melanosis.*—In the Autumn of 1835, a young woman applied to Dr. Warren, with a tumor on the anterior part of the thigh, about the size of a hen's egg. It had existed five or six months, and no cause for it had been discovered. When the muscles were relaxed, it was freely moveable in a lateral direction; when they were contracted, it was perfectly fixed. It was free from pain or tenderness. Soon afterwards, Dr. W. removed it. An incision being made over the rectus muscle, its tendinous fibres were exposed below the middle of the thigh. These being separated, the tumor was found buried in the midst of the rectus, from which it was removed with considerable pain and hæmorrhage. The wound united in part by the first intention; but a small sinus remained open about four weeks, after which she recovered, and has had no return. On examination of this tumor, it proved to be a melanosis. It was seen to be composed principally of hard dark-coloured muscular substance; but its nucleus consisted of an osseous shell, an inch in diameter, containing a dark-coloured fluid, and lined by a black crust.

Dr. Warren offers a solution of the formation of the tumor. The patient, he conjectures, received a blow on the part, which ruptured some vessels. A coagulum was formed, and gradually covered with an osseous crust. The injured muscular substance might become indurated either before or after the formation of the crust, and partook of the melanotic action. He adds that the case was certainly not malignant, as the patient has been well for two years. We cannot subscribe to so positive a declaration. We apprehend that the records of surgery will furnish many instances in which as long a period as two years has elapsed without a recurrence of malignant tumors after operations, and yet the patient has ultimately had such a recurrence, and died of it.

2. *Scirrhous Muscular Tumor.*—We only allude to this for the purpose of noticing a case in which Dr. Warren operated. Why we do so, will presently be evident.

*Case.*—A shoemaker had a tumor of three years' growth, of which the following is the account given. Beginning at the upper edge of the cartilage of the fifth rib, it extended to the lower edge of that of the eighth, and required the breadth of six fingers to cover it vertically, and of four to cover it transversely. Its breadth lay from the median line to the left, beginning from the linea alba. In colour, the skin was not changed, excepting that it exhibited numerous enlarged veins. In consistence it had the hardness of a periosteal tumor, that is, something less than that of bone. A degree of sensibility existed on the edge of the cartilage of the fifth rib, and at some other points. It was slightly moveable in a lateral direction, but not in the vertical, and its movement did not appear to affect the ribs. There was a sensible pulsation, without any vibration.

All the circumstances made it probable that this tumor was of a scirrhous character, and far from improbable that it was adherent to the peritoneum. Dr. Warren determined to remove the tumor, which he did on the 9th of March, 1837.

"The patient being placed on a table, an incision, seven inches long, was made from the fourth rib downwards, and the anterior face of the tumour was exposed by dissecting away the integuments. Its face being laid bare, exhibited a blueish colour, and was of a scirrhous hardness. Every stroke of the knife around it was followed by a copious flow of blood. When the circumference of the tumour was uncovered, its edges were found to be quite undefined and concealed by the muscular connexions. These being cut through, I was able to discover an ill-defined edge, and proceeded with the knife under this edge, and soon found I could detach the tumour from the faces of the cartilages; pursuing the dissection altogether by the touch, as the streams of blood and the hardness of the tumour prevented an inspection of the parts beneath. The operation proceeded favourably, till the tumour turned down over the cartilages into the epigastric region. Here it was obvious that if it involved the internal oblique and transversalis to the extent it did the external oblique and rectus, the incisions would penetrate the cavity of the peritoneum, especially if this membrane adhered to the inner face of the tumour. Such was found to be the fact. The two inner muscles formed part of the disease; the peritoneum adhered to it for about an inch, and not having the advantage of ocular inspection, it was of course difficult by feeling to make a nice dissection of the adherent membrane. This was, however, accomplished, and the whole disease was removed in one mass." 70.

Smart fever succeeded the operation, and slight peritoneal inflammation demanded two or three bleedings. The wound healed in the third week.

On examining the tumor, it presented a cartilaginous hardness. Its surface on all sides, was composed of the muscles between which it lay. Its substance consisted of a brownish texture, in which a multitude of granulations, the sixteenth of an inch in size presented, with intermixture of a fibrous arrangement. At one point there was a softening, as if suppuration were about to begin. At another a dark red spot was seen. The internal or epigastric part was equally hard with the rest of the tumor. The surface of the cartilages was deeply depressed where the tumor had lain.

We allude to this case, partly because the situation of the tumor renders it interesting, but chiefly because we think it dubious whether an operation was proper. In the first place, the tumor had all the characters of scirrhus —any operation would therefore be likely to fail in the long run; in the second place, the connexions of the tumor were uncertain, and it was probable, as it really proved, that it was attached to the peritoneum, if it did not extend into the chest—circumstances which would render an operation hazardous in its immediate consequences, and still less likely to be favourable in its ultimate. With such objections to the employment of the knife, we believe that the majority of well-informed surgeons in this country would decline operating, and we believe too, that they would be right in their refusal. A surgeon should have strong grounds for anticipating final success, before he subjects his patient to a painful process, which may itself destroy him. He should weigh not only the heavy responsibility of the life of a fellow-creature being brought by his aid into jeopardy, but the effect on the public mind of severe operations lightly engaged in, and disastrously terminated. The whole profession, humanity itself, have an interest in seeing that operations are not abused. One unsuccessful case may create terror in the minds of many persons, and be the means of preventing the performance of operations that would not be unsuccessful. We have again and again urged this consideration. We have denounced unnecessary and injudicious operations, and we will continue to denounce them. We make no account of the interested clamour that has been raised, and probably will still be raised against us. That is of little moment. We are pleading the cause of humanity. Not that we accuse Dr. Warren of trifling in this instance with the life or the feelings of another. He acted for the best, and, though we differ from him, we do so with respect. But there are persons whose motives are more questionable than those of Dr. Warren—whose science is much less—and whose temerity is much greater. Let them beware, for public feeling is gathering against them.

On malignant melanosis there is nothing to be said, nor on fungus hæmatodes. But a case is related as singular, and an enumeration of its main features and result may not be useless.

*Case.* In 1827, a lady, between 30 and 40 years of age, consulted Dr. W. on account of a tumor, on the outer and upper part of the left thigh, below the trochanter major. It had existed for some months, and had been preceded by jaundice. There was an irregular hardness in the part mentioned, for about the extent of a hand's breadth ; presenting much the feeling of the swelling in œdematous erythema ; but not discoloured, and something harder than the swelling mentioned. It was not tender to the touch, nor painful. The general health somewhat impaired. The lady was under the necessity of being removed to New York. From that city, the following account of the tumor was received.

" The tumour, as you will remember, is not well defined on either of its margins, but is gradually lost in, and apparently incorporated with the natural parts—Of the hardness of ordinary scirrhous tumours—and evincing little sensibility on pressure—though there is considerable irritation and soreness immediately above and below. There is a point of the tumour which has been gradually becoming more prominent, and over which the skin is already tense and discoloured—yet the hardness there is scarcely less than when Mrs. — first arrived here. The pain in the back, hip, and down the limb would be oftentimes intolerable, but for the recurrence to strong anodynes ; indeed Mrs. — has rarely a comfortable hour without their aid. Above and below the tumour there is considerable swelling—sometimes more, and sometimes less—and within a few days, œdema about the lower part of the spine and sacrum. When Mrs. —

arrived here, one of the inguinal glands, on the side affected, was much enlarged, prominent and very hard. This has been increasing, so as now to project more than an inch—but shows no disposition to suppuration." 86.

The tumour increased in size and became more pointed, while the skin grew thinner, and finally ulcerated, giving issue to a small quantity of serous discharge. Before the patient's death, which was the effect of exhaustion from pain, &c. a very extensive and hard tumor was discovered in the abdomen.

On dissection, the glands of the abdomen were found extensively diseased. The liver was indurated, and the other abdominal organs were more or less the subjects of scirrhous disorganizations. The primary seat of the tumor in the thigh, which, no doubt, was of a scirrhous nature, does not seem to have been made out.

3. *Tumors of the Tendons.*—Our author's remarks on this head contain no novelty; but he relates a case which deserves attention.

*Case.* A clerk, 18 years old, extremely muscular, in the Summer of 1834 undertook to raise himself from the ground, while sitting with his lower extremities extended; and this was to be done without using his hands. This feat he succeeding in accomplishing. No immediate inconvenience was recollected. Two or three weeks after this act, he perceived a weakness about the knees, and examining, found a slight hardness along the tendons of the flexor muscles. He continued to move about, but found the tumors and lameness regularly increasing. Three months after the exertion alluded to, he consulted Dr. Warren. On examining him, there was felt along the tendons of the biceps muscles, in both limbs, a hardness of about four inches in length, and near an inch in thickness, principally seated on the inner edge of the tendon, and partly on its posterior face. When the muscles were contracted by an effort to raise the leg, the patient lying on his face, the tumor became of a stony hardness. When the muscles were relaxed, it was less obvious. No distinct uneasiness was caused by compressing the part. At this period, he was unable to walk more than a few steps.

It appeared that the young man had been under the care of "a bone and sinew doctor."* Dr. Warren enjoined a severe discipline. He informed the patient that he must prepare himself for a confinement of at least a year, possibly for two years or more. He was directed to keep in a horizontal posture on a bed; the knees to be slightly flexed, and supported in this posture, and never to be moved, excepting passively. Twelve to eighteen leeches every fifth day for the first six weeks. Occasionally a purgative. No animal food, and the quantity of food limited. The sequel of the case may be given in our author's words.

* Much as John Bull is given to quackery, it would seem that his American cousin is still more so. Such personages as "bone doctors" are not to be discovered now in any town of size in England. In some rural districts, where intelligence is longest in penetrating, the bone-setter may still be found. Yet even there, he languishes, and is only indifferently supported by the ignorance and credulity of the lower part of the rustic population.

"I heard no more from him for five months. He then sent for me. I found him pale and emaciated. He had severe pains in the bowels, and frequent bleedings at the nose. He had followed the course directed in every particular. I expressed my surprise that he should have undertaken to proceed so long a time without giving me an opportunity of accommodating the treatment to circumstances. He apologised by intimating, that being compelled to relinquish his occupation, he was without means of defraying more than the expenses of subsistence. From this time, I attended him regularly. I should have mentioned that the tumours were sensibly diminished. He was now directed to take a little meat twice a day—to drink from four to six ounces of wine, and 40 drops of tincture of sulphate of quinine every two hours. Whenever the abdominal pains were urgent, to take 30 drops of tincture of opium. Under this management, he recruited slowly; and at the end of four weeks the wine gave him headache, and was discontinued. Animal food was continued a month longer, and then seeming to disagree with him, was omitted. He now resumed the original course, with the exception of substituting injections for purgatives, and sometimes taking a little meat. After eight months' confinement, he was directed to use frictions of a stimulating liniment, and to have the limbs bathed twice a day in hot water and soap. Finding the tumour lessening regularly, when he had been confined a year, I directed him to bandage the whole limb, and to get out of bed once a day. This was followed, after two or three weeks' trial, with a thickening of the cellular substance between the hamstring muscles; in consequence of which he was put on the bed for a month longer, and then advised to get up and walk the room once a day. At the end of 15 months, he was able to walk a quarter of an hour at once. I now urged him to walk out; but his apprehension of reproducing the disease was so strong, that it was some-time before this could be effected. At last he got out, and increasing his walks very gradually, in three months he was able to walk to my house; and I had the satisfaction to find that all remains of the swellings were wholly dispersed. Since then, he has recovered his strength, and is now perfectly well." 94.

We suppose there can be little doubt that this was an instance of chronic inflammation of the tendon, or its sheath, or both. There is no evidence of any specific character having belonged to the tumor; and the progress and issue of the case are both opposed to such a notion. We cannot help considering Dr. Warren's treatment as rather, perhaps unnecessarily, severe. Had local depletion and counter-irritation been actively employed, we think that the rigorous regimen and absolute confinement might have been much mitigated. Such heroic treatment will, in many constitutions, induce diseases more serious than the local malady for which they have been instituted.

Dr. Warren relates a case of what he denominates fungus non hæmatodes. He does not appear to us to be sufficiently aware of what the experience of late years has satisfactorily determined, that the hæmatoid character of some fungous tumors is merely an accidental one, dependent on the general state of the individual, and on the existence of accidental blood-vessels of some dimensions. The medullary sarcoma, encephaloid tumor, and fungus hæmatodes, are only varieties of the same description of morbid growth, all being equally malignant, all having a disposition to fungate, and all, when fungating, having more or less of a tendency to venous hæmorrhage. We need not enter on the considerations which go to establish this conclusion. It is sufficient for us, at present, to observe, that, in the minds of most pathologists, it is established.

Dr. Warren, too, would appear to have scarcely arrived at the practical inferences with respect to these tumors, which may be said to be received as principles in this country. We have seen him operating on a scirrhous tumor in the abdominal parietes under circumstances which were highly unfavourable. In a case of fungoid tumor, growing from above the right knee, and extending from the superior edge of the patella upwards, for about three inches, we find the following operation resorted to.

"Finding there a deep-seated tumour, not discoloured, I was quite at a loss to determine what it was. One thing was clear, that it must be removed. The operation was performed in June, 1834. After making a free incision of the skin and cellular texture, I sought for the tumour. Instead of it, I found the extensor tendon of the muscles of the thigh expanded over the surface, and no way was discovered of getting under it. Of course, I divided the tendon longitudinally, and then came in contact with a white fungus more firm than fungus hæmatodes, but breaking in pieces when handled, and showing no disposition to bleed. Continuing the dissection, I found this fungus intermixed with fair looking fasciculi of tendinous substance, much interwoven with it, but of a totally different aspect from the tumour. Having followed its irregularities into the interstices of the neighbouring muscles, by a laborious dissection, I at length got out the whole." 97.

Now we think that many surgeons would have suspected that the tumor was of a fungoid character, and proposed amputation, rather than dissecting the tumor out. But whether they suspected that or not, we are pretty sure that they would have immediately decided on urging amputation, when they discovered that it *was* a fungoid tumor, and that it was disseminated in the contiguous muscles. Under such circumstances the satisfactory extirpation of the tumor became next to impossible, and the only reasonable chance of cure was from the removal of the limb.

It is now a canon in surgery, that under the most favourable circumstances which can be anticipated, the removal of really malignant tumors is any thing but a successful operation. The disease in a great proportion of cases appears in some other part of the body at some future time. This is found to be the fact when the complete extirpation of the morbid growth can be ensured—as in castration—the removal of the mamma—amputation of the affected limb. If such an operation, apparently so effectual, is unfortunate, à fortiori a less effectual one will be so. Hence the rule—to operate as early as possible—and, when a limb is the seat of the disease, to amputate. Hence, also, the rule not to extirpate fungoid tumors under circumstances which render their perfect removal precarious. We dwell on these points because it is, obviously, of the greatest consequence to the community that operations should be regulated by the best principles that our science enables us to arrive at.

If we refer to Dr. Warren's case we shall find, that, so far as one case can do so, it bears out what we have urged. The lady on whom he operated, re-applied on to him in the Autumn of 1835, rather more than a year after the operation. The tumor had returned, regained nearly its original size, and was growing rapidly. She now refused amputation. Had this measure been sternly insisted on in the first instance, no delusive confidence would have been engendered in any other measure, and she *might* have assented, *might* have been saved. But this by the way. She desired a second removal of the tumor, and Dr. Warren consented. This operation nearly proved fatal. But she recovered from it and appeared to be cured. In the Autumn of 1836, the thigh again began to swell, a tumor formed in the groin, another tumor was felt in the abdomen, *it* filled with water, and probably ere this the poor lady's sufferings have been relieved by death.

### V. Periosteal Tumors.

Dr. Warren makes some remarks on periosteal tumors, but they cannot be considered as complete. The following case of much thickening of the lower jaw from necrosis is worth reading.

*Case.* In 1835, a sea-faring man applied to Dr. Warren, on account of a tumor on his face. This was situated on the right side, extending from the edge of the lower jaw to the zygomatic arch, and had somewhat the form, and nearly the size of a lemon. It had existed a year; came on without any known cause; was colourless; quite hard; not attended with much pain, and made a formidable appearance. The patient's health was perfectly good. The disease had been suspected to be exostosis; osteo-sarcoma; fungus of the antrum. Dr. W. examined the mouth, and at length discovered a small aperture, leading to the lower jaw, a probe introduced into which struck on rough bone.

He was at once satisfied of the nature of the case. He made a perpendicular incision about three inches long, and laid bare the masseter; after dissecting through this muscle a semi-cartilaginous substance appeared; the knife with difficulty penetrated this, and being forced to some depth did not reach any thing of a different character. He then proceeded to excise this hard substance; and neglecting the circumference, cut out a mass an inch and a half square and an inch thick, from its centre. This being removed, a probe was passed, and struck a diseased bone. The aperture was then enlarged, a strong forceps introduced, and with some difficulty a dead piece of the lower jaw was extracted, about two inches long, and comprising the whole mass of the lower jaw-bone for that extent. The wound slowly healed, the remainder of the hard substance was absorbed, and, in three weeks after the operation, the patient was discharged.

The next case may be quoted, chiefly on account of the singularity of the operation successfully performed for its cure.

*Case.* A gentleman, aged 40, had an abscess form on the right side of the chest, at the junction of the bone, and cartilage of the sixth rib, in consequence of a typhus fever, which occurred in the Summer of 1834. In the Spring of 1835, Dr. Warren first saw the patient. There was an aperture about an inch deep at the part indicated, surrounded by an irregular hard substance for two or three inches. The probe after passing down an inch struck on a firm but not osseous substance, and could not be made to proceed deeper by altering its direction. The discharge of pus from the wound was small. In the succeeding Winter the pain increased about the ribs, and there was occasionally troublesome hiccough. The patient now made up his mind to undergo an operation, the dangers of which were fully explained to him. The operation may be described in the operator's own words.

"The aperture through which the probe passed, was opposite the junction of the cartilage of the sixth rib, with its bone. It was about the sixth of an inch in diameter, and surrounded by a firm substance, which I at first supposed to be thickened and hardened cellular membrane. The plan which I formed for the operation was this. To make an incision four inches long, over the sixth rib, passing across the aperture. To cross each extremity of this wound with another, two inches long, at right angles to it. To dissect up the two flaps, expose the ribs, divide the intercostal muscles, and fascia, separate the pleura and diaphragm from the inner face of the ribs, and saw or cut through them. As happens in most operations, the execution varied considerably from the plan. An incision was made four inches long over the bone and cartilage of the sixth rib, and one at each extremity of the first, about two inches long. Proceeding to raise the flaps as proposed, I here met an obstacle. The skin adhered to a hard substance under it, so closely, that it was extremely difficult to raise the integuments, without cutting them in pieces. This being at length accomplished, the had substance presented itself, covering the ribs and cartilages, and confounding the appearance of the bones, and cartilages in a mass of semi-ossified fibro-cartilage ; so that I was at a loss how to prosecute the operation, in order to discover the seat of the diseased bone or cartilage. I proceeded in the following manner— The semi ossified matter was shaved off in successive layers, till it became so thin as to allow a strong director to be pushed in under this substance, and under the rib with which it was cemented; and passing it from within outwards, and from above downwards, for about two inches, between the pleura and the ribs, with this director for a guide, other portions of the hard matter were removed, till the diseased ribs and cartilages were sufficiently uncovered. About three inches of the bone and cartilage of the seventh rib, and somewhat less of the sixth were carefully separated from the pleura, and diaphragm, and cut out by the chain saw, and cutting forceps." 107.

No artery, of consequence, was wounded. A smart pleuritic attack occurred on the second day, but was checked by a copious bleeding. Subsequently, from over exertion, inflammation ensued below the wound, and suppuration was the consequence, succeeded by much induration and a fistulous opening. But the patient recovered from all, and is now quite well and strong.

It must be owned that the operation was a difficult and dangerous one, and its termination more fortunate than could be counted on.

### VI. OSSEOUS TUMORS.

Dr. Warren adopts Sir A. Cooper's division of exostoses into the periosteal and medullary. It does not appear necessary to follow him in his observations, but we may pick out a remark or two.

A. In describing an operation for the removal of a large portion of the lower jaw, he offers the following hints on the construction of saws for the purpose.

"The saw employed was constructed for the purpose, and had the following dimensions: Length, two inches; breadth, two lines; thickness, one-fourth of a line; handle, four inches long, rough and thick; end of the saw rounded. This is the best kind of saw that can be employed for this and similar operations. The chain saw and the bow saw cut more rapidly, but catch and cause delay and embarrassment. Heys' saw is slow and laborious in operation. The rotatory saws are unmanageable. I think a saw half an inch longer, that is, two and a half inches long, might have been better. The great difficulty in the use of the saw, in these cases, has led me to describe this simple instrument minutely, with the hope that it may be as useful to others as it has been to me. It is proper to have two or three of these saws, varying a little in dimensions." 114.

B. A man applied to Dr. Warren's father with an exostosis of the right cornu of the os hyoides, of a sugar-loaf form, about three inches in height. His father dissected the tumor to the os hyoides; exposed this bone, sawed it off near its base; and thus succeeded in curing the patient speedily and effectually.

Neither the observations nor the cases advanced under the head of medullary exostosis require more than mere mention. The next species of exostosis alluded to, is—*Maxillary Exostosis of the Spongy Texture.* The account which our author gives of this disease is brief.

"The spongy texture of the jaws, especially of the upper, is subject to inflammation and exostosis. Tumours of this kind are without much difficulty distinguished from periosteal exostosis of the jaws by this; that they are swellings of the *substance* of the bone, and not of its *surface.* The last is most commonly seen on the lower jaw; the first on the upper. Exostosis of the spongy texture of the jaw, is a hard tumour, of a rounded form; appearing on the external surface of the bone; producing a prominence of the gum. and if it attains a considerable size, of the cheek. It is not discoloured, nor painful. Enlarges rapidly at first in young subjects. This disease often begins in the bottom of an alveolus. A diseased action arises in the membrane, is propagated to the bone which swells in the outward or peripheral direction, commonly in the earlier stage of the disease, though it happens sometimes at the internal plate of the jaw becomes involved ultimately." 127.

The tumor which our author describes is not malignant. It constitutes, in fact, that sort of tumor which may be, and which continually is, removed from the upper jaw, with a considerable degree of success. Our author relates several cases in which he operated and effected a permanent cure. But there is not sufficient novelty in such cases to induce us to extract them.

The propriety of ascertaining whether abnormal conditions of the teeth may not have given rise to these maxillary tumors, as well as kept them up when formed, has been frequently insisted on in lectures and in books. The advice is so important, that we are tempted to cite two cases of our author's that tend in a striking manner to enforce it.

*Case* 1. Some years since, Dr. W. was desired to see a young man, for a large sugar loaf shaped tumor of the cheek, of some months growth, perfectly hard and insensible. On cutting into it, a tooth was discovered in the maxillary cavity: after the removal of which, the bony tumor soon disappeared, and the cheek resumed its natural form.

*Case* 2. "In the year 1828, I was called to see a young lady, eight years old, who had a considerable tumour on the left side of the face. It was noticed three months before; had since increased much. It was very hard; not painful nor tender. No blow had been received on the part, and no cause was known for its existence. The tumour being in the situation of the maxillary cavity, and the process of dentition being at that time in activity, I suspected a tooth had slipped into the cavity, and advised not to do any thing at the time; and after watching the tumour for some months, seeing no increase, and nothing that could lead to the suspicion of malignity, I ceased to attend her, and heard no more of the matter until May, 1836. Her mother then informed me, that four weeks previous, a tooth had appeared on the inside of the upper jaw, within the other teeth; and as soon as this happened, the tumour in the face began to lessen. The tooth was extracted; since which the relicks of the swelling had been rapidly disappearing. The patient being now a fine handsome young lady of sixteen, felt very grateful to me for having spared her a bad scar, by cutting open the antrum." 133.

The next case is of rather a different description. Yet it, too, shews the influence exerted by the teeth in the production of maxillary tumors.

*Case.* A young lady, aged 28, of a nervous and scrofulous constitution, had the right superior cuspidatus tooth filled in 1834. About three months afterwards, she had a severe pain in this and in the bicuspid tooth behind it, which continued, with occasional remissions, until April, 1836, when she applied to Dr. Warren. When he examined the jaw, he found the cuspidatus tooth slightly loosened; the last incisor, next to it, very loose; a red tumor extending over the alveoli of these two teeth, and in a slight degree over that of the next incisor. No swelling within the alveoli. The surface of the gum presented an unhealthy ulceration, which extended to the membrane of the cheek.

Dr. W. prescribed regimen, laxatives, and cinchona as a local application, without benefit. The ulceration increased, and Dr. W. thought it only prudent to remove the morbid structure. First, he took out the cuspidatus and the incisor teeth. Next surrounded the ulcerated part with an incision. Then with perpendicular cutting forceps divided the bone on each side, and by a horizontal forceps connected the two incisors, and removed the diseased bone. The whole operation was accomplished in three or four minutes. In December, 1836, the patient paid our author a visit. The jaw was quite sound, and the lost teeth had been replaced by artificial ones.

We may now turn to some practical observations on the mode of operating. They deserve attention.

"In the early periods of my practice, I removed the bone in these cases with the saw and chisel, and the operation was tedious and painful. In the year 1831, I first employed the cutting forceps, by which this operation was greatly facilitated. Since then, the ingenuity of my friend, Dr. Flagg, to which I have often been indebted, has greatly improved this instrument, in its application to the jaws. As the straight forceps are with difficulty brought to bear uniformly on the inside and outside of the jaw, he gave to these instruments the hawk's bill form. One of these is made to cut vertically, and another horizontally; by which the two kinds of incisions are rapidly and perfectly made; and the patient suffers but little pain. The introduction of the cutting forceps into the surgery of the jaws, is a great practical improvement. Instead of the long and painful operations with the saw, they give us the power of removing a bony tumour from these parts in a few minutes. When the tumour is of some extent, they have not sufficient power. This defect may be remedied by a compound forceps, which bring in the aid of an additional lever."* 135.

*Osteo-Sarcoma.* This is a very indefinite term, and has been applied in an equally indefinite sense. Vague as are our ideas with regard to the nature,

---

* "Since the above was written, I have seen a pair of cutting forceps, from France, which cut at right angles from their shaft, and must be very useful, when the tumour lies far back in the mouth."

the forms, and the discrimination of the tumors of soft parts, they are pre-eminently so when we arrive at tumors of the fibrous and the osseous tissues. We know of no respectable account of the various morbid growths from periosteum and bone, an account which at the same time gives a good particular, and a philosophical general view of them. Such an account is a desideratum, and Dr. Warren's plan, founded on mere local considerations, is inconsistent with it. We must, therefore, take his remarks on osteo-sarcoma as they are proferred—a clinical report on the more remarkable facts that he has seen. The following is the description of the growth which he offers.

"This is a hard tumour, taking its origin from periosteum or bone. In external appearance, it has usually a firm consistence, a rounded form, extends over and appears to involve and bury the bone, from which it is derived. In colour it is like the skin commonly, though sometimes reddened, and exhibiting enlarged veins, like many other tumours of similar consistence.

Although hard in its early stage, as it increases in size, it acquires more or less elasticity, and of course softens as it grows. The consistence is, in fact, variable from day to day; at least in some instances. Whether this accidental change is caused by the different quantities of blood it may contain, or by a change in the amount of the fluids deposited in its cavity, I am unable to say. The size also has appeared to be slightly variable; but that such fluctuations do actually exist, seems questionable. A variation in the colour of the integument is not surprising; as the prominence of the part exposes it to friction, and the temporary increase of morbid action in the interior would affect the external covering.

The pain is not acute. A sense of aching is a common, though not invariable attendant.

A characteristic symptom of osteo-sarcoma is a tuberculated surface. The inequalities which cause this appearance are not produced by an intervention of plates of bony and cartilaginous substance. They are the masses of the unequally-thickened periosteum, which form the covering of the tumour. In osteo-sarcoma I have never seen bony plates excepting at the points where the tumour is connected with the bone. If, for example, the bone be cylindrical, bony fragments may be noticed at those parts where the extremities of the bone enter the tumour. At other points I have not seen them." 137.

Osteo-sarcoma is, as Dr. Warren observes, very generally believed to arise from the reticular structure of bone. But in all the cases which he has seen, it has appeared to have its origin in the periosteum. The constituents of the tumor, according to his view of it, are three. 1. The bone on which the tumor is placed. 2. The periosteum. 3. The medullary fungus, contained in the periosteum. To these we must add a fluid of varying consistence, found in cells, in the interior of the periosteal cavity.

"First; the bone in most of these cases appears as if a part of it had been broken out, and the fractured ends are enlarged, or else the thickened periosteum gives them the appearance of enlargement. But this appearance is not universal. You may see in the case of Corey, the third case mentioned, that the rib from which the tumour originated was entire. Its surface only, shewed the origin of the tumour. Consequently, the tumour in this case must have had its origin in the surface; and this fact cannot be considered as a mere exception to a general law; it breaks up such a law; for if a true osteo-medullary tumour can be traced to a periosteal origin in one instance, we may believe it to have such an origin in others, unless the contrary is proved. Second; the periosteum in these tumours exhibits an extraordinary activity of growth. It is quite vascular, and forming the surface of the tumour, is thickened in some parts to the extent of half an inch, and forms the protuberances characteristic of the tumour, as you may verify in this tumour of the clavicle and that of the rib. Third ; the fungoid formation which constitutes the mass of the tumour, appears, so far as we are able to discover, to arise from the periosteum nearest the bone, whence it shoots in a pedunculated or arborescent form into the large periosteal cavity. So much for this short survey of the anatomical structure." 139.

It does not appear to us that Dr. Warren's reasoning proves his position. According to his experience, what he terms osteo-sarcoma originates in the periosteum. To support his statement, he refers to cases in which it seemed to do so. So far so good. But that does not prove that, in other cases, a similar tumour may not originate in the reticular texture of bone. We have seen cases in which it clearly did so, and had we leisure, we have no sort of doubt that, in a very few hours, we could cite recorded cases in which there was every proof, that can be obtained, of the same thing.

The fact is, that osteo-sarcoma, as our author has defined it, is nothing more than fungoid tumor, modified by the tissue from which it springs. Now, we know too well that this form of morbid growth may be developed, and is constantly developed in every tissue. Why, then, attempt to limit it to one? Why affect to determine that it only grows from periosteum or from the cancellated texture of bone ? It grows from either, and these exclusive views have no foundation in nature, nor in a liberal reading of facts.

Dr. Warren attempts to point out a distinction between "medullary exostosis" and osteo-sarcoma. Such distinctions are only verbal. The wider our acquaintance with the morbid growths become, the less faith is attached to such nice discriminations. What one man chooses to call medullary exostosis, and to endow with certain characteristics, appears on paper to differ from what another terms osteo-sarcoma, and endows also with peculiar characters. But, in practice, cases continually occur which we are at a loss to refer to either the one category or the other, but which have the marks of both. We have seen, for instance, two cases of what may either be termed medullary exostosis, or osteo-sarcoma, growing from the cranial diploë. The evidence of that was perfect. If the characters mentioned by Dr. Warren are to be deemed conclusive of the presence of osteo-sarcoma, both cases were osteo-sarcoma; but then in both, the cancelli of the cranial bones were the seat of the disease. The truth is, that modern experience has gone some way in determining two things with reference to the morbid growths ;—first, that many of the appearances that constitute varieties are deceptive, they not being sufficiently uniform, nor permanent; and, secondly, that our means of discriminating the actual varieties are unsatisfactory and unworthy of much confidence. What, for instance, can be more uncertain than diagnosis founded on such data as the following?

"How may this be distinguished from other tumours ? From exostosis it may be known by wanting the hardness of the latter. From common periosteal tumour, by its greater size and more regular form. From scirrhus, situated on a bone, by being more closely connected with the bone. From medullary exostosis, in wanting the basket-like surface, caused by mixture of layers of bone and membrane. The history of its growth will aid in illustrating the distinctions: yet, after all, there are cases in which it is impossible to ascertain with certainty what its nature is." 144.

The admission in the last paragraph eats up the value of the signs in its predecessors; and that admission is most true.

The only rules of practice at which, in the present condition of our knowledge, we are enabled to arrive are these:—*that*, those tumors which succeed or are accompanied by impaired constitutional powers, and which seem of unequal consistence, being soft in some parts and hard in others, are probably of a malignant character—*that* if there is an appreciable disposition to fungate, this probability is much increased, and if there have previously been, or are, other growths in other parts, of a malignant character, the probability may be said to be almost converted to a certainty—*that*, whatever the character of the growth may be, if it is clearly going on to mischief, it should be removed by operation, supposing, of course, that there are no peculiar local or general circumstances to prevent a resort to the knife—*that*, if the disease be pretty ceriainty malignant it should be removed, unless it extends so far as to interfere with a perfect removal, or unless there are reasons for believing that internal organs are implicated—*that*, if tumors of a fungoid, or, perhaps, if even of a common scirrhous character, be situated in the limbs, amputation, as the most effectual operation, should, cæteris paribus, be preferred to extirpation of the growth—*that*, under the most favourable circumstances, the fungoid morbid growths most frequently, and the scirrhous frequently, reappear, after operations, in some part of the body, and at an uncertain time—this tendency to reappearance being proportionally augmented by imperfect, late, or improper operations—*that*, our means of diagnosis, though generally accurate, are occasionally fallacious; tumors that seem *not* malignant returning after operations, and tumors that *do* seem malignant not returning—and, finally, *that*, the sum of these considerations appears to amount to little more than this:—our means of discriminating these tumors being imperfect, and our practice being founded on a vague calculation of mere probabilities, we are bound to lean to the side of hope, and to *offer* a patient the chances of an operation when it is not forbidden by some positive consideration. We say to offer a patient these chances, not to press them on him. The case we think should be stated fairly, or with a very little leaning towards encouragement. It is one thing to keep up a patient's hopes while suffering from disease, a practice which is one of our strongest remedies; but it is another thing to seduce him by fallacious assurances into encountering a painful operation, which will probably be worse than useless, because it will bring surgery itself into discredit. The profession, we say once more, is interested in putting down unnecessary operations. Ignorance, temerity, or the most brutal sense of self-interest, may make an individual reckless of high considerations of this kind, but, as a benevolent and a scientific body we are bound to denounce and to frustrate such heartlessness.

Dr. Warren relates three cases. The first is one of osteo-sarcoma of the clavicle, for which that bone was extirpated. We cannot say that we approve, under the circumstances, of the operation, but, as the operation itself must be deemed one of the greatest in surgery, it is only justice to Dr. Warren to furnish our English readers with an account of it. There can be no doubt that he presents a good specimen of a bold and dexterous surgeon.

*Case.* D. Smith, aged 24, farmer, admitted into the Massachusetts General Hospital, Nov. 1, 1832.

One year previously, in attempting to roll a heavy stick of timber over, he placed both arms under it, bringing his chest firmly against it at the same time. Immediately after it he felt a severe pain and an enlargement near the junction of the right clavicle with the sternum. After finishing his work, he applied to a physician for advice. The physician did not understand the case, but the swelling was lessened by warm bitter fomentations.—Continued to work, though his arm was quite weak, till the month of August last, when he quitted his labour, thinking he had "sprained the other collar bone." The following month, October, the swelling began to increase rapidly, and became hard. At times he felt, as he thought, a "rubbing of the bones."

Now he has a tumour measuring seven inches from sternal end of the right clavicle, in a line with this bone to the scapular end; from its upper boundary, viz. the clavicle, towards the nipple, five inches. Thinks it varies in size, being sometimes larger that at others. At times feels it pressing on the wind-pipe; it feels hard; there is no evident fluctuation; a pulsation is slightly perceived in it by stethoscope; some pains most of time; increased by using arm, or by coughing; pulse eighty-four; no sensible difference in the pulsation at wrists; general health not materially impaired, but constitution scrophulous and irritable.

On the 10th of November, the operation of removing the clavicle was performed. The steps pursued were the following:—

"The patient being placed on a table, the shoulders elevated, an incision was made from the acromial extremity of the clavicle to the sternal extremity of the clavicle of the opposite side. This was crossed by an incision at right angles with it, beginning just below the middle of the sterno-mastoid muscle, and extending to the face of the pectoralis muscle below the middle of the clavicle. The four flaps were then dissected from the surface of the tumour. Next, the outer extremity of the clavicle was laid bare, by dissecting the deltoid muscle from its anterior edge, and the trapezius from its posterior edge, and by the division of the coraco-clavicular ligaments. An eyed probe, armed with a ligature, was then passed under the clavicle, and the ligature being attached to a chain saw, this was drawn under, and the clavicle sawed through.

The separation of the tumour now began. A strong ligature being passed around the outer extremity of the divided clavicle, the tumour was partially raised by it, so as to give tension to the surrounding soft parts. The pectoralis major muscle was cut through, and dissected from the lower edge of the tumour, and drawn so as to expose the pectoralis minor and the cephalic vein. Now the dissection extending under the tumour, the subclavian muscle was distinguished and dissected from the tumour at its outer part, but at the sternal part it was lost in the tumour, where, of course, the dissection proceeded, over the surface of the subclavian vein. An adhesion of the tumour to the second rib, in which it was imbedded, prevented a perfect separation of this part until at the close of the operation.

The next step was to divide the attachments of the upper or cervical edge of the tumour. First, the posterior external jugular vein was divided and tied. It was filled below with a dense lymph, and discharged some red blood from above. Next, the sterno-mastoid muscle was cut across, and the sheath of the cervical blood vessels exposed. The internal jugular vein was perceived to pass from the neck into the substance of the tumour, in such a way as to render it difficult to dissect it without dividing it. This was, however, accomplished, and the carotid artery and par-vagum nerve presented themselves. On reaching the internal extremity of the tumour, the anterior external jugular vein was found bedded in it. This was filled with solid lymph, and was tied, as it would not have been safe to have left it without a ligature. Nothing now remained to be done, but to separate the sternal end of the tumour from the corresponding part of the jugular and subclavian veins. By great caution this was safely accomplished, and the tumour removed. When this was effected, the whole extent of the subclavian, the lower part of the jugular vein and par-vagum nerve were exposed.

These being put in motion by the pulsation of the subclavian and the carotid arteries, presented a formidable appearance.

Little blood was lost in the operation. Only one or two arteries were tied, and the veins specified above. The flaps were brought over and retained by three sutures and adhesive plasters, so as to cover the wound perfectly." 150.

This was, no doubt, a very formidable operation, and, no doubt, it was performed with great skill and great determination. The patient went on well until the thirteenth day, when "he was affected with chills," and pain in the epigastric region. The pulse rose from 80 to 112, "a nervous agitated state" succeeded, and, eventually, a slight delirium. On some days he appeared better, and in a fair way to recover; and this hope was not abandoned till the day before his death, which occurred on the 8th of December, in the fourth week from the operation.

*Dissection.* "A slight adhesion appeared at the point where the pleura corresponded with the wound; but this membrane, in the vicinity of the wound, was sound, and the lung not inflamed at that part. The inferior edge of the right lung was fringed with lymph. The lung of the other side had a more considerable effusion of lymph externally, and *in its substance two or three spots of effused lymph, near an inch in diameter.* A quantity of sanguineous fluid was discharged into the left cavity. The heart and pericardium were unchanged. The brain presented nothing remarkable. The viscera of the cavity of the abdomen were healthy, excepting a slight blush where the intestines were in contact, and an enlargement of the right kidney. The subclavian vein, the upper part of the axillary, and the cephalic veins, each contained a coagulum, which was of long standing, and adhered closely to the coats of the vessels." 152.

Dr. Warren mentions that three years subsequently he was informed by a person who had been a patient in the hospital along with this person, that he caught his fatal chill by leaving his room to pay a visit, when his nurse was absent. We would observe, however, that the affection which proved fatal to this patient was not mere inflammation of the lungs, but the "secondary deposites." The "spots of effused lymph in the substance of the right lung, near an inch in diameter," are too characteristic to permit us to entertain a doubt on this head; while the "chills," or rigors, succeeded by quick pulse, a peculiar state of agitation, and symptoms that appeared so vague and fluctuating as to lead those unacquainted with the affection to anticipate recovery up to the very day before death, are quite as characteristic as the pathological appearances.

Now as these secondary affections constantly occur independently of any exposure to cold, and seem rather to depend on the kind of operation performed, than on any accidental circumstances of that sort, we must hesitate before we attach much importance to the individual's having quitted his room. Of all operations, those in which incisions are carried into the deep cellular membrane, and those in which veins are much meddled with, appear to be most frequently succeeded by the secondary inflammations and deposites. In the present case both circumstances obtained. Large veins were cut, or tied, or exposed, and the cellular membrane under the deep cervical fasia must have been more or less interfered with. We are somewhat surprised at Dr. Warren's making no allusion to this common and fatal consequence of operations. Perhaps his attention has been little directed to it—perhaps it may not be as common in Massachusetts as it is in the large cities of Europe. It is only within the last 15 or 20 years that we have become acquainted with it, on this side of the Atlantic.

Here our limits compel us to stop for the present. Our ample extracts will evince our sense of the practical interest which attaches to the present work. It is essentially a clinical report on tumors, and has the merit which such reports possess, of presenting objects as much like naturė as the signs of language can represent them. Its facts deserve to be perused by all, but especially by those who have not the advantage of hospital experience. To their charge tumors usually fall in their commencement, when sound opinions and judicious practice are of most importance. The bulk of the profession must necessarily, in some matters, have imperfect experience, such experience being only attainable where cases are presented in masses, as in hospitals. To supply, so far as the press can supply, the deficiency so felt by numbers of our brethren, has ever been, and ever will be our object. So long as we exist as journalists, our aim will be the diffusion of what we believe to be useful knowledge.

We shall return to this work in our next number. In the mean time, we return our thanks to Dr. Warren for enabling us to bring his labours under the notice of the profession in Great Britain. We are most anxious to cement the scientific union between the cousins of America and England.

